# Bibliometric analysis and visualization of transdermal drug delivery research in the last decade: global research trends and hotspots

**DOI:** 10.3389/fphar.2023.1173251

**Published:** 2023-06-16

**Authors:** Xinghan Chen, Haitao Xiao, Xiujun Shi, Qiao Zhao, Xuewen Xu, Ping Fan, Dongqin Xiao

**Affiliations:** ^1^ Research Institute of Tissue Engineering and Stem Cells, Nanchong Central Hospital, The Second Clinical College of North Sichuan Medical College, Nanchong, Sichuan, China; ^2^ Department of Burns and Plastic Surgery, West China Hospital Sichuan University, Chengdu, Sichuan, China; ^3^ Department of Pharmacy, West China Hospital Sichuan University, Chengdu, Sichuan, China

**Keywords:** transdermal delivery, hotspot, bibliometric analysis, biclustering analysis, visualization

## Abstract

**Background:** Transdermal delivery has become a crucial field in pharmaceutical research. There has been a proliferation of innovative methods for transdermal drug delivery. In recent years, the number of publications regarding transdermal drug delivery has been rising rapidly. To investigate the current research trends and hotspots in transdermal drug delivery, a comprehensive bibliometric analysis was performed.

**Methods:** An extensive literature review was conducted to gather information on transdermal drug delivery that had been published between 2003 and 2022. The articles were obtained from the Web of Science (WOS) and the National Center for Biotechnology Information (NCBI) databases. Subsequently, the collected data underwent analysis and visualization using a variety of software tools. This approach enables a deeper exploration of the hotspots and emerging trends within this particular research domain.

**Results:** The results showed that the number of articles published on transdermal delivery has increased steadily over the years, with a total of 2,555 articles being analyzed. The most frequently cited articles were related to the optimization of drug delivery and the use of nanotechnology in transdermal drug delivery. The most active countries in the field of transdermal delivery research were the China, United States, and India. Furthermore, the hotspots over the past 2 decades were identified (e.g., drug therapy, drug delivery, and pharmaceutical preparations and drug design). The shift in research focus reflects an increasing emphasis on drug delivery and control release, rather than simply absorption and penetration, and suggests a growing interest in engineering approaches to transdermal drug delivery.

**Conclusion:** This study provided a comprehensive overview of transdermal delivery research. The research indicated that transdermal delivery would be a rapidly evolving field with many opportunities for future research and development. Moreover, this bibliometric analysis will help researchers gain insights into transdermal drug delivery research’s hotspots and trends accurately and quickly.

## 1 Introduction

Transdermal delivery administration is a method of administering drugs via the skin (Prausnitz et al., 2004). Since the approval of the first scopolamine transdermal patch in 1979 (Prausnitz and Langer, 2008), it has become a widely utilized technique in medicine due to its safety, painlessness, self-administration ease and ability to bypass degradation by digestive tract enzymes (Ita, 2017; Wang et al., 2018). The skin is an important barrier that protects the body from the external environment and prevents the entry of harmful substances (Chouhan et al., 2019). However, it also presents a challenge for the delivery of drugs, as the skin’s properties make it difficult for drugs to penetrate and reach their intended target (Hiraishi et al., 2013). The transdermal drug delivery is developing to overcome this challenge and provide a convenient, non-invasive, and safe method of drug delivery (An and Liu, 2017; Akhtar et al., 2020; Defraeye et al., 2020; Shabbir et al., 2020).

The transdermal drug delivery has many advantages over traditional oral and intravenous administration. For example, it can provide sustained release of drugs over a longer period of time, reducing the need for frequent dosing (Zhang et al., 2019; Longo et al., 2021; Shankar et al., 2021; Saeedi et al., 2022; Yang et al., 2022). This can be particularly beneficial for chronic conditions that require long-term treatment, as it can help to improve patient compliance and reduce the risk of side effects (Iyer et al., 2022; Jimenez-Rodriguez et al., 2022). Transdermal drug delivery can also help to bypass first-pass metabolism, which can reduce the potential for drug interactions and improve the bioavailability of the drug (Kassem et al., 2018; Raviraj et al., 2021; Zhao et al., 2022a; Zhang et al., 2022). Furthermore, transdermal delivery can avoid the potential for gastric irritation, ulceration, and other gastrointestinal adverse effects that are often associated with oral drug administration (Ita, 2015; Zhang et al., 2020).

Despite the promising developments in transdermal delivery, there are still several challenges that need to be addressed. The skin is composed of several layers, each with different physical and chemical properties, and drugs must penetrate these layers to reach their intended target, which can be a major challenge in transdermal delivery research (Ita, 2016a; Amjadi et al., 2017; Oyelaja et al., 2020). Factors such as the size and charge of the drug, the presence of skin-irritating excipients, and the occlusive properties of the delivery system can all impact the permeation of drugs through the skin (Villasmil-Sanchez et al., 2010; Duangjit et al., 2013; Qin et al., 2013; Shi et al., 2014; Rullier-Birat et al., 2015). Accordingly, the development of transdermal delivery systems requires a multidisciplinary approach, incorporating expertise from a variety of fields including dermatology, pharmacology, chemical engineering, and material science (Ahmad et al., 2021; Sirbubalo et al., 2021).

Bibliometrics is a quantitative approach to analyzing and evaluating scientific literature through the use of statistical methods and visualization techniques (Joyce et al., 2014). This field of study enables the identification of research hotspots and trends within a particular domain, providing valuable insights into the current state of the field and guiding future research efforts. For example, through bibliometric analysis, researchers have been able to determine the current state of research on topics such as microneedles and biological wound dressings (Chen et al., 2022a). By objectively revealing research trends, bibliometrics helps to inform and guide the direction of future research endeavors.

This study aims to conduct a comprehensive evaluation of the existing literature on transdermal drug delivery through a bibliometric analysis approach. The objective is to provide a deeper understanding of the field of transdermal drug delivery research by utilizing co-word biclustering analysis. The results of this study are expected to aid researchers in gaining a more efficient and accurate understanding of transdermal drug delivery research trends and developments.

## 2 Methods

### 2.1 Data source and search approaches

**FIGURE 1:**
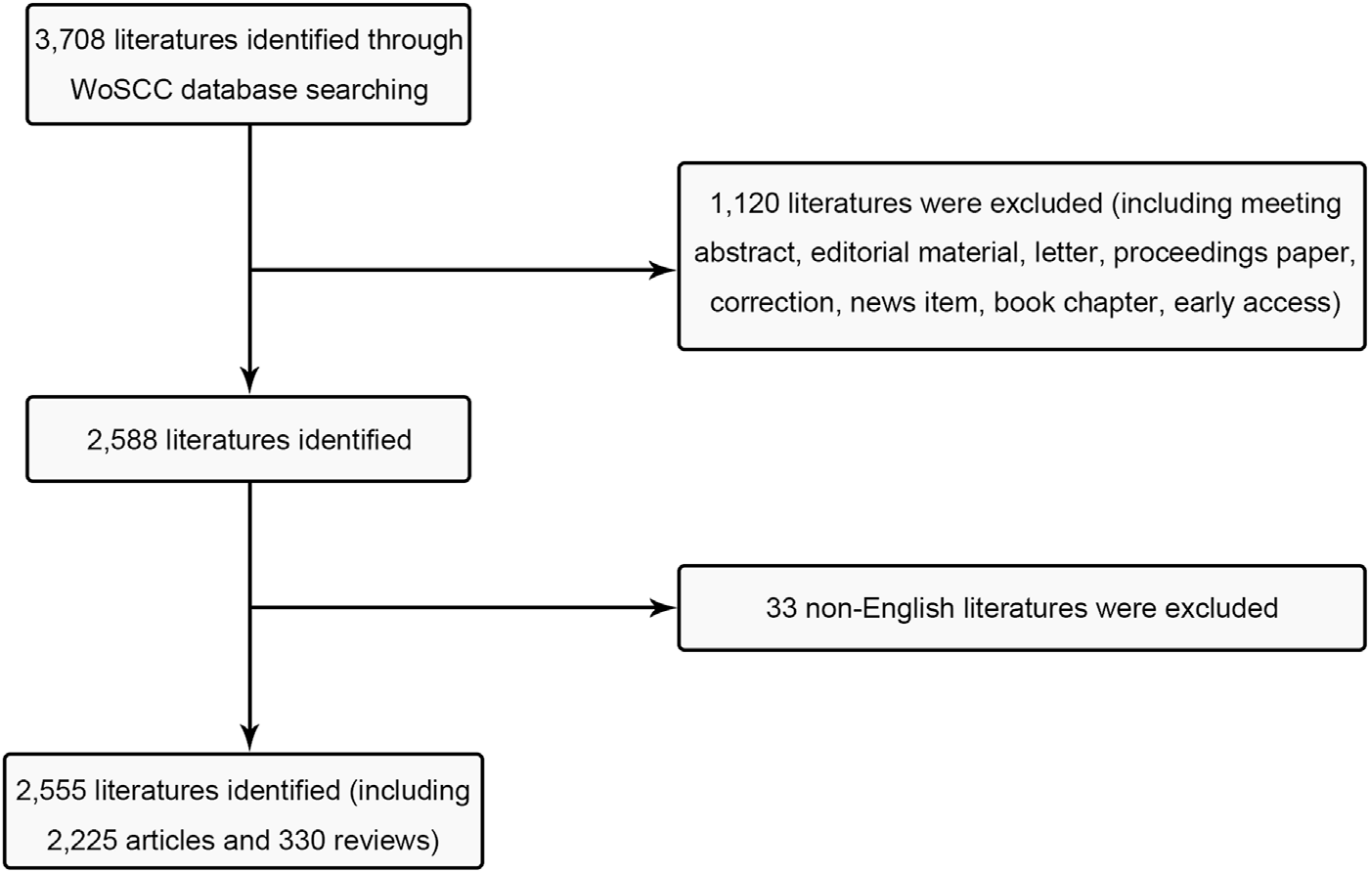
Flow chart of inclusion and exclusion.

Initially, the Science Citation Index Extension (SCI-E) within WOS was utilized, following these search criteria: TI = (Transdermal delivery) and language = English, with a specified time frame spanning from 1 January 2003, to 31 December 2022. A total of 3,708 studies were collected, and then some literature types were excluded including i) meeting abstract; ii) editorial material; iii) letter iv) proceedings paper; v) correction; vi) news item; vii) book chapter (viii) early access (Figure 1). Additionally, non-English literature was deliberately omitted. Subsequently, the co-word clustering analysis will be conducted using the Medical Subject Heading (MeSH) terms retrieval feature provided by the NCBI. The time range for the analysis was set from 2003 to 2022, with “Transdermal delivery” designated as the MeSH term. To prevent potential biases stemming from database updates, all publications were retrieved on 11 December 2022.

### 2.2 Data collection

Two reviewers independently assessed the search results, including title, publication date, country/region, author information, citation count, and H-index. The overall agreement ratio between the reviewers was calculated to be 0.90. In cases there were conflicting evaluations, a third reviewer was involved to determine whether to include the respective data.

The data imported into the online analysis platform of Bibliometrics (http://bibliometric.com/) and VOSviewer V1.6.17 (Leiden University, Leiden, the Netherlands) was in the “Tab Delimited File” format. For CiteSpace V 6.1.R4 (Drexel University, Philadelphia, PA, United States), the imported data format was “Plain Text File”. The MeSH terms retrieved from NCBI were formatted as “PubMed” and input into BICOMB V2.02 for analysis. Subsequently, the co-word matrix file generated from the analysis was utilized for biclustering visualization using gCLUTO V1.0 (Graphical Clustering Toolkit).

## 3 Data investigation

### 3.1 Bibliometric analysis and geographical distribution

The online analysis platform of Bibliometrics was used to visualize international collaborations and national/regional contributions. VOSviewer was employed to generate a colorful clustering visualization based on the inter-agency cooperation degree among institutions. Additionally, density clustering visualization was based on the number of published journals. When utilizing VOSviewer, the data format selected was “Create a map based on bibliographic data”, with “Co-authorship” chosen as the type of analysis, resulting in the creation of the “Density Visualization” graphic. Moreover, CiteSpace was employed to identify time-dependent burst words, visualize research trends, and predict future research directions. After importing the data into CiteSpace, it is essential to utilize the “Remove duplicates” function before setting “keyword” as node types and selecting “The number of states” as 2. The Journal Citation Report (JCR) published in 2022 was consulted to ascertain the journal impact factors (IF).

### 3.2 Co-word biclustering analysis

BICOMB and gCLUTO were utilized to conduct biclustering analysis on major MeSH terms and MeSH subheadings, aiming to identify research hotspots. In BICOMB, a “PubMed-2” format file was created, and the corresponding data of MeSH terms were imported. The “Extract” function was employed to set “main topic + sub-topic”. Following this, the major MeSH terms were transformed into a matrix using software, and a co-word matrix of high-frequency MeSH words was generated. Subsequently, the co-word matrix was imported into gCLUTO, where the “Number of Clusters” value was specified, and the “Cluster Method” function was set to “Repeated Bisection”. Lastly, the matrix and mountain visualization were employed to present the results of biclustering analysis. Through this biclustering analysis, valuable insights regarding the research hotspots in transdermal delivery can be obtained.

## 4 Results

### 4.1 Investigation of publications output

**FIGURE 2:**
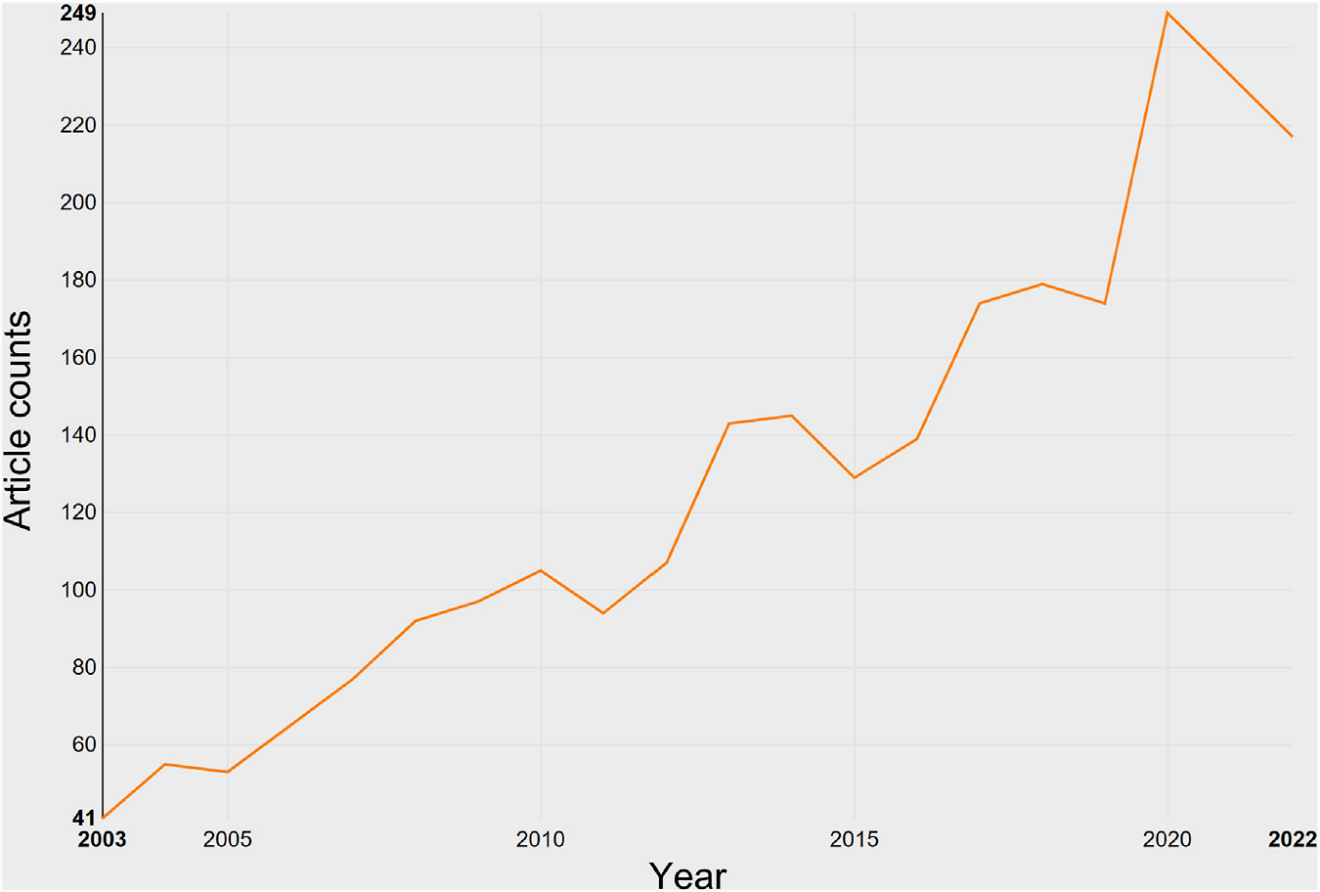
Growth of publications on transdermal drug delivery (2003–2022).

In total, 2,225 studies and 330 reviews meeting the inclusion criteria were identified in the field of transdermal delivery (Figure 1). From 2003 to 2022, there was a consistent increase in the number of publications, with a notable surge in 2020 (Figure 2). As of now, 176 studies have been published in 2022, which is more than six times the number published in 2003. However, it is important to note that the total number of publications in 2022 is expected to be higher than 176.

### 4.2 The contributions of nations and institutions to global publications

**FIGURE 3:**
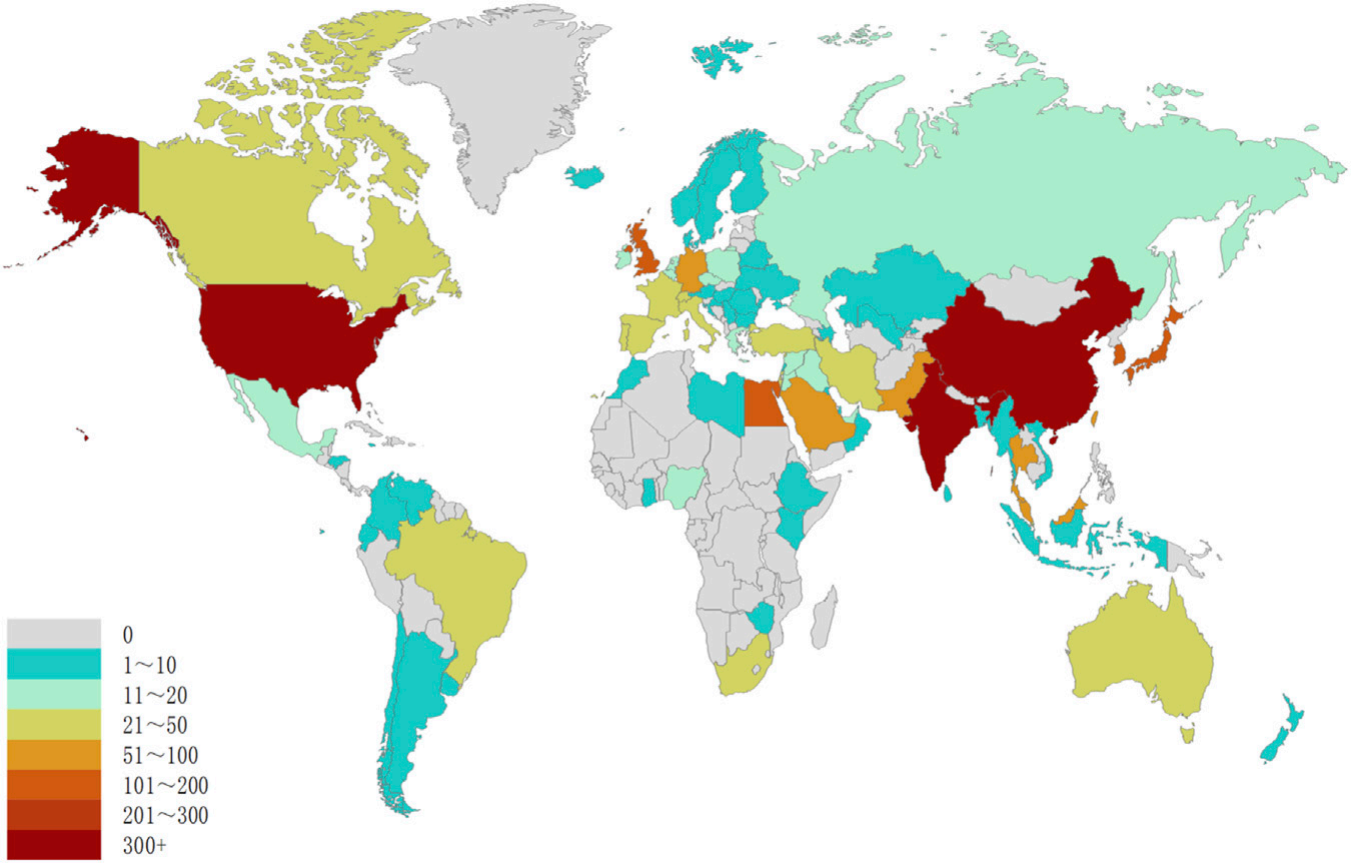
Geographical distribution of retrieved articles in transdermal drug delivery (2003–2022).

**FIGURE 4:**
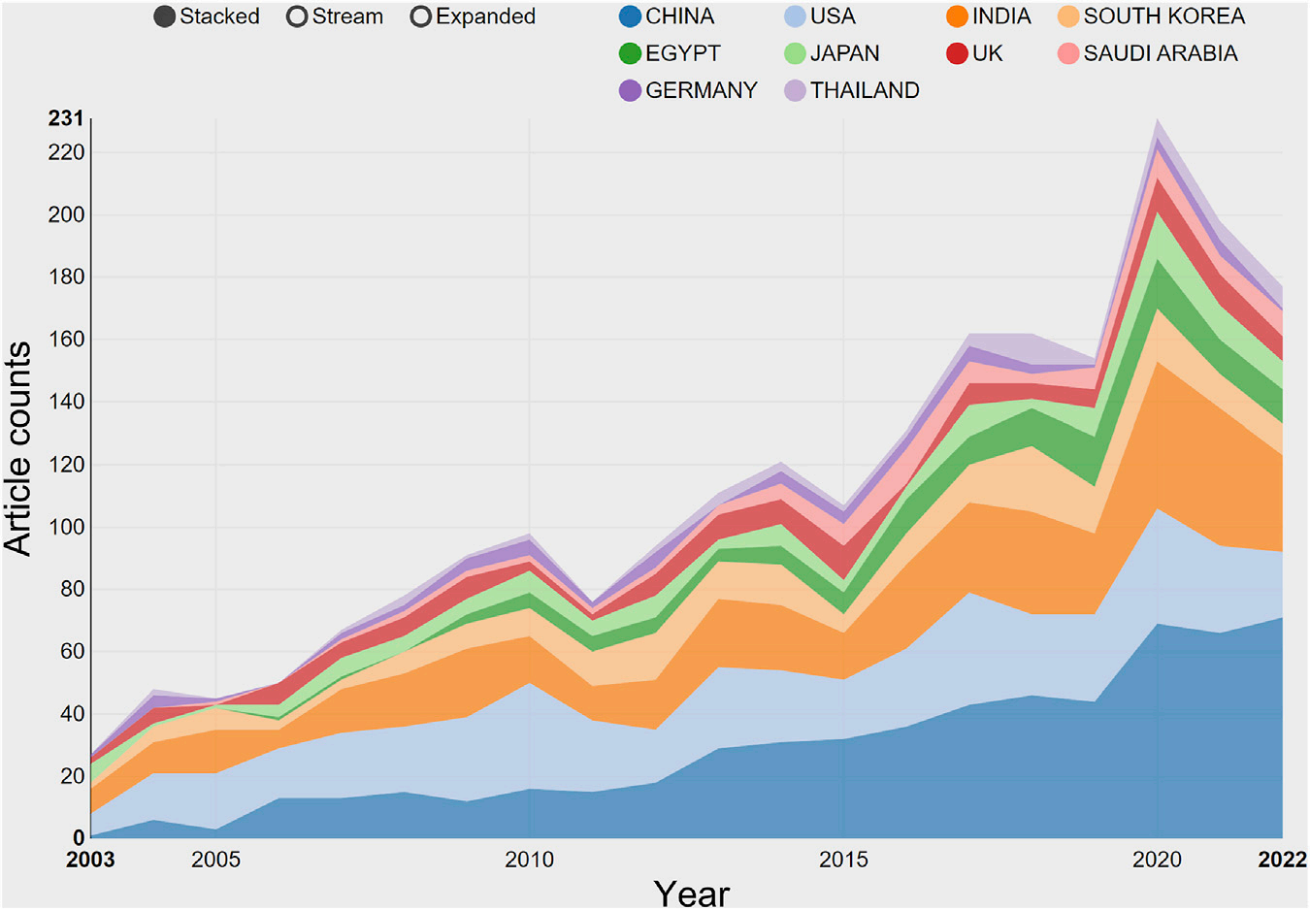
The growth trends of the top 10 nations/regions in transdermal drug delivery (2003–2022).

**FIGURE 5:**
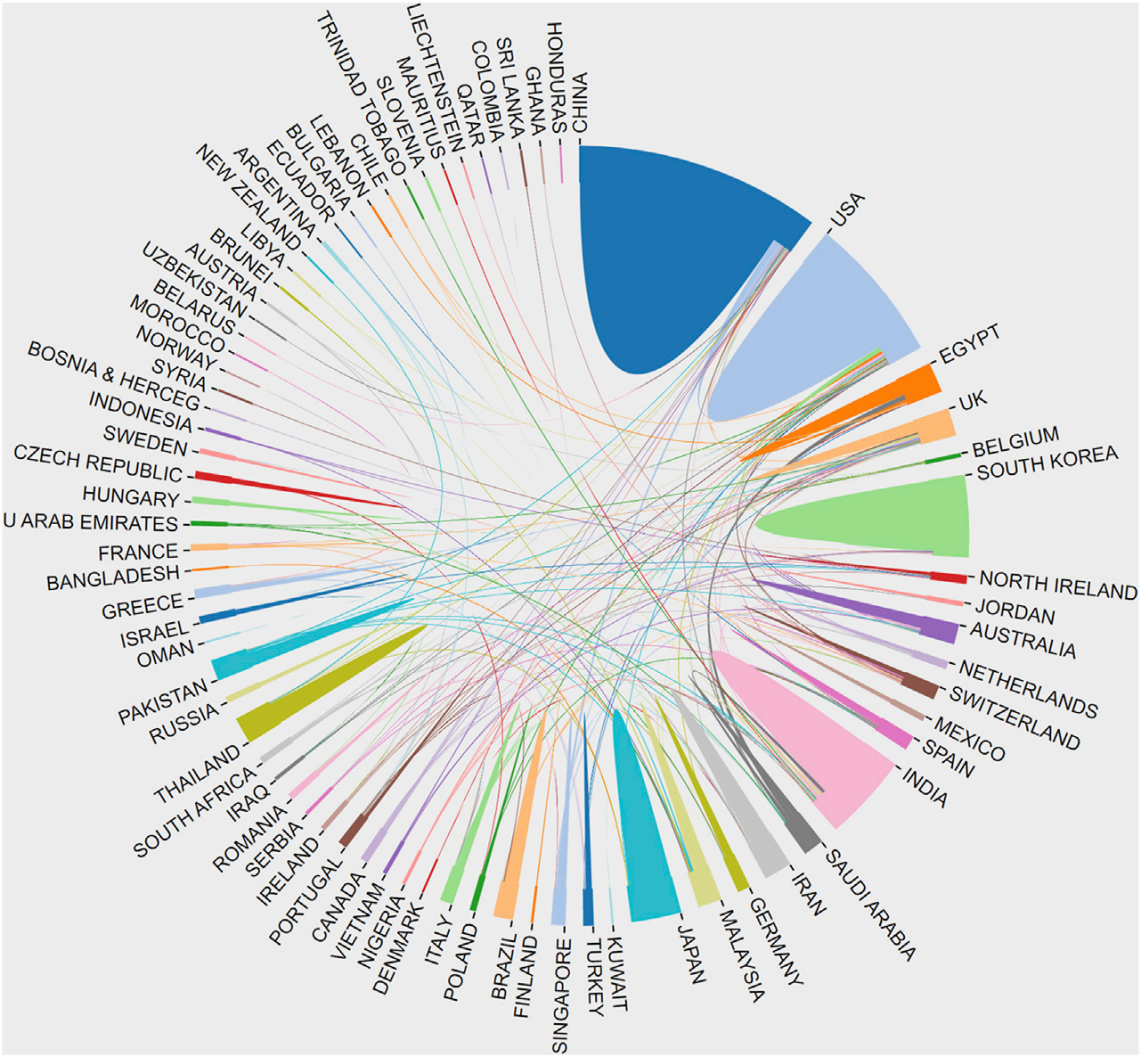
The network map of country/region’ cooperation.

**FIGURE 6:**
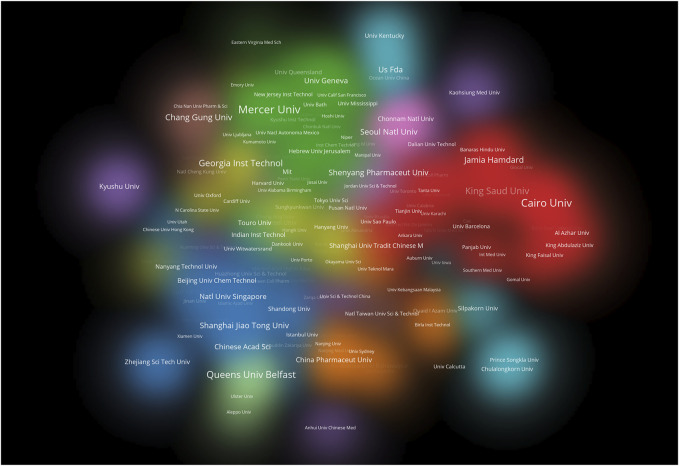
Cluster density visualization map of institutions on transdermal drug delivery (2003–2022).

The studies on transdermal delivery originated from 89 distinct countries or regions. Upon importing the data, the thermal world map (Figure 3) revealed that these studies were primarily concentrated in East and South Asia, North America, and Western Europe. Notably, China (*n* = 491) emerged as the primary contributor, followed by the United States (*n* = 474) and India (*n* = 431) (Table 1). The growth trajectory of published literature in this field for major countries is depicted in Figure 4. A survey on national/regional collaborations indicated that China and the United States engage in the most frequent cooperation (Figure 5). Centrality serves as a crucial indicator of a country’s involvement in international collaboration, with higher centrality suggesting greater influence on others. The findings reveal that the United States exhibits the most prominent centrality (center = 0.37), followed by India (0.34) and the UK (0.28). For research institutions, the top five were Egyptian Knowledge Bank (*n* = 124), Jamia Hamdard University (*n* = 52), Mercer University (*n* = 48), Cairo University (*n* = 46), and Queens University Belfast (*n* = 43) (Table 1). Utilizing VOSviewer, inter-agency collaborations were examined based on co-authorship, and the findings were visualized as density clusters (Figure 6). The analysis of inter-agency partnerships revealed the division of agencies into nine distinct clusters, each represented by a different color.

**TABLE 1:** Top ten productive country/region and institutions on transdermal drug delivery(2003–2022).

Rank	Country/region	Article counts	Centrality	Total number of citations	Average number of citations	Institutions	Article counts	Total number of citations
1	China	491	0.13	11,155	22.72	Egyptian Knowledge Bank	124	3,230
2	United States	474	0.37	21,847	46.09	Jamia Hamdard University	52	1,775
3	India	431	0.34	11,589	26.89	Mercer University	48	1,405
4	South Korea	184	0.05	6,082	33.05	Cairo University	46	1,430
5	UK	121	0.28	3,934	32.51	Queens University Belfast	43	3,066
6	Egypt	125	0.07	3,239	25.91	University of California System	42	3,325
7	Saudi Arabia	79	0.03	2,090	26.46	University System of Georgia	40	8,401
8	Thailand	58	0.02	1,062	18.31	King Saud University	38	1,371
9	Germany	57	0.08	2,188	38.39	Chinese Academy of Sciences	33	1,483
10	Pakistan	54	0.04	632	11.7	Chang Gung University	31	1,052

### 4.3 Journals publishing research on transdermal drug delivery

**FIGURE 7:**
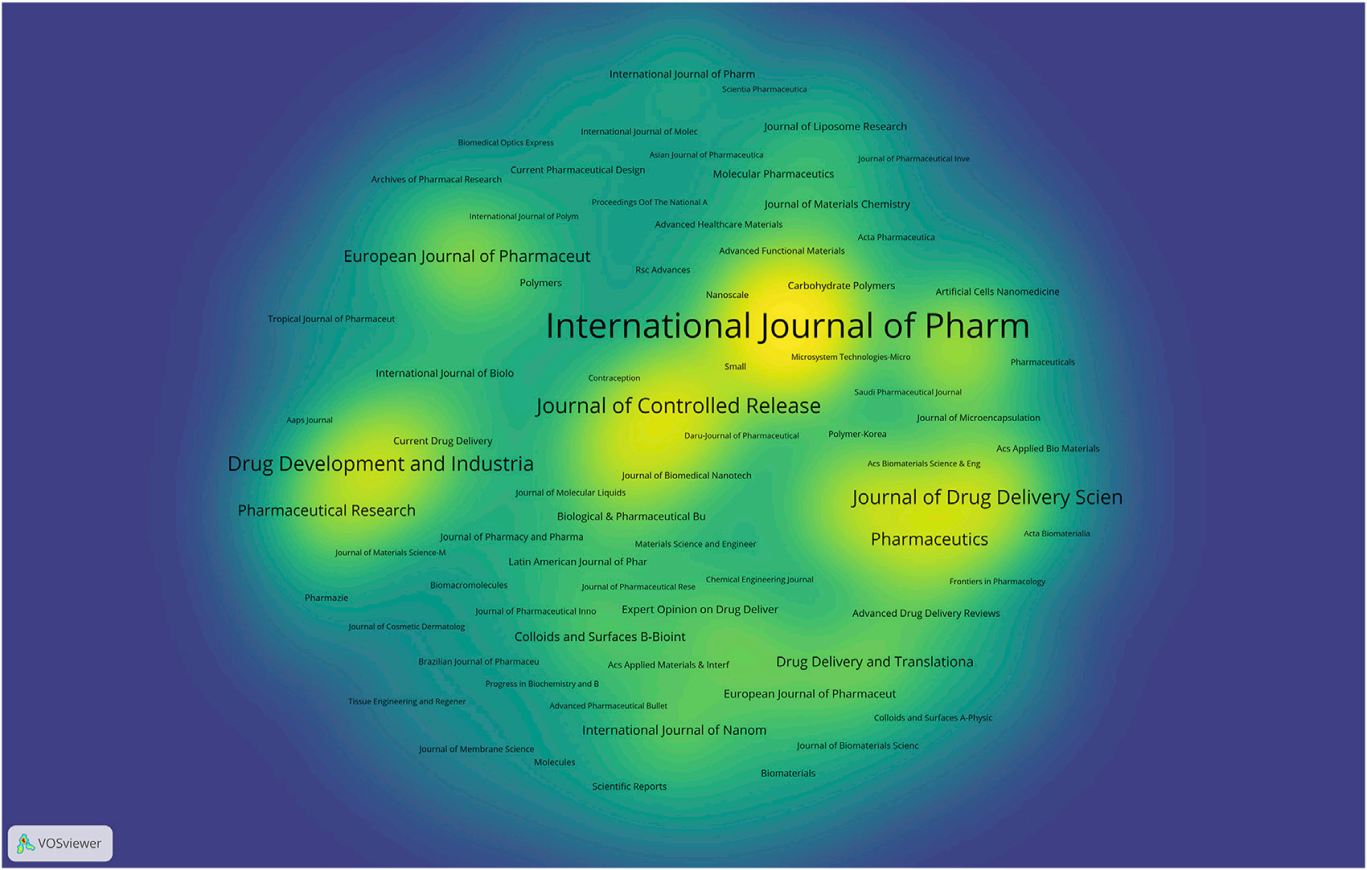
Density visualization map of journals on transdermal drug delivery (2003–2022).

A total of 576 journals have contributed to the publication of studies on transdermal delivery, and Figure 7 displays the journals that have made notable contributions to this research field. Of all 2,555 studies of transdermal delivery, 878 (34.36%) were published in the top 10 journals (Table 2). The top three journals include International Journal of Pharmaceutics, Journal of Controlled Release, and Drug Development and Industrial, accounting for 15.15% of all the included studies. Among the journals that published more than 10 studies, Advanced Functional Materials (IF: 19.924) stood out with the highest impact factor, followed by Advanced Drug Delivery Reviews (IF: 17.873) and Biomaterials (IF: 15.304). Importantly, all three journals hold the Q1 classification according to the JCR 2022 standard.

**TABLE 2:** Top ten journals in the publication on transdermal drug delivery (2003–2022).

Rank	Journal	Article counts	Percentage (N = 2,555) (%)	IF (2022)	H-index	Total number of citations	Average number of citations
1	International Journal of Pharmaceutics	200	7.828	6.510	51	7,858	39.29
2	Journal of Controlled Release	98	3.836	11.467	48	8,055	82.19
3	Drug Development and Industrial Pharmacy	89	3.483	3.727	26	2,073	23.29
4	Journal of Drug Delivery Science and Technology	87	3.405	5.062	18	1,085	12.47
5	Drug Delivery	76	2.975	6.819	25	2,095	27.57
6	Aaps Pharmscitech	75	2.935	4.026	25	2,006	26.75
7	Journal of Pharmaceutical Sciences	72	2.818	3.784	22	1,697	23.57
8	Pharmaceutics	67	2.622	6.525	17	1,451	21.66
9	European Journal of Pharmaceutics and Biopharmaceutics	59	2.309	5.589	32	3,038	51.49
10	Pharmaceutical Research	55	2.153	4.580	27	2,300	41.82

### 4.4 Authors’ contributions to transdermal drug delivery research

In this study, a total of 8,232 authors contributed, and Table 3 presents the top 10 productive researchers among them. Banga, Ajay K. from Department of Pharmacology & Pharmacy, Mercer University, Macon, Georgia, United States. Donnelly, Ryan F. from Department of Centre Medical Biology, Queens University Belfast, Belfast, Antrim, North Ireland. Prausnitz, Mark R. from Department of Biomolecular Engineering, Georgia Institute of Technology, Atlanta, Georgia, United States. An analysis was conducted to identify studies with a high citation frequency, and the top 10 studies in the field of transdermal delivery are presented. The most frequently cited study was " Transdermal drug delivery " published by Prausnitz, MR et al. in Advances in Advanced Drug Delivery Reviews in 2008 (*n* = 1,858) (Table 4).

**TABLE 3:** The top ten most productive authors contributed to publications in transdermal drug delivery research.

Rank	Author	Article counts	H-index	Total number of citations	Average number of citations
1	Banga AK	45	20	1,324	29.42
2	Donnelly RF	42	27	3,066	73
3	Prausnitz MR	29	24	8,095	279.14
4	Aqil M	26	18	1,024	39.38
5	Zhang Y	25	15	759	30.36
6	Goto M	21	11	501	23.86
7	Kalia YN	21	17	684	32.57
8	Jiang GH	20	14	731	36.55
9	Stinchcomb AL	19	13	467	24.58
10	Ahad A	18	16	720	40

**TABLE 4:** Top ten cited articles on transdermal drug delivery (2003–2022).

Rank	Title	Journal	Corresponding author	Publication year	Total citations
1	Transdermal drug delivery	Advanced drug delivery reviews	Prausnitz, MR	2008	1,858
2	Microneedles for transdermal drug delivery	Nature reviews drug discovery	Prausnitz, MR	2004	954
3	Current status and future potential of transdermal drug delivery	Journal of controlled release	Prausnitz, MR	2004	939
4	Biodegradable polymer microneedles: Fabrication, mechanics and transdermal drug delivery	Science advances	Prausnitz, MR	2005	629
5	Wearable/disposable sweat-based glucose monitoring device with multistage transdermal drug delivery module	Proceedings of the national academy of sciences of the United States	Hyeon, T	2017	608
6	Microfabricated needles for transdermal delivery of macromolecules and nanoparticles: Fabrication methods and transport studies	Biomaterials	Prausnitz, MR	2003	567
7	Dissolving microneedles for transdermal drug delivery	Advances in colloid and interface science	Prausnitz, MR	2008	560
8	Microemulsions as transdermal drug delivery vehicles	Advanced drug delivery reviews	Garti, N	2006	500
9	Transdermal skin delivery: Predictions for humans from *in vivo*, *ex vivo* and animal models	Journal of controlled release	Touitou, E	2007	485
10	Coated microneedles for transdermal delivery	Nature biotechnology	Prausnitz, MR	2007	455

### 4.5 Research hotspots of transdermal drug delivery

**FIGURE 8:**
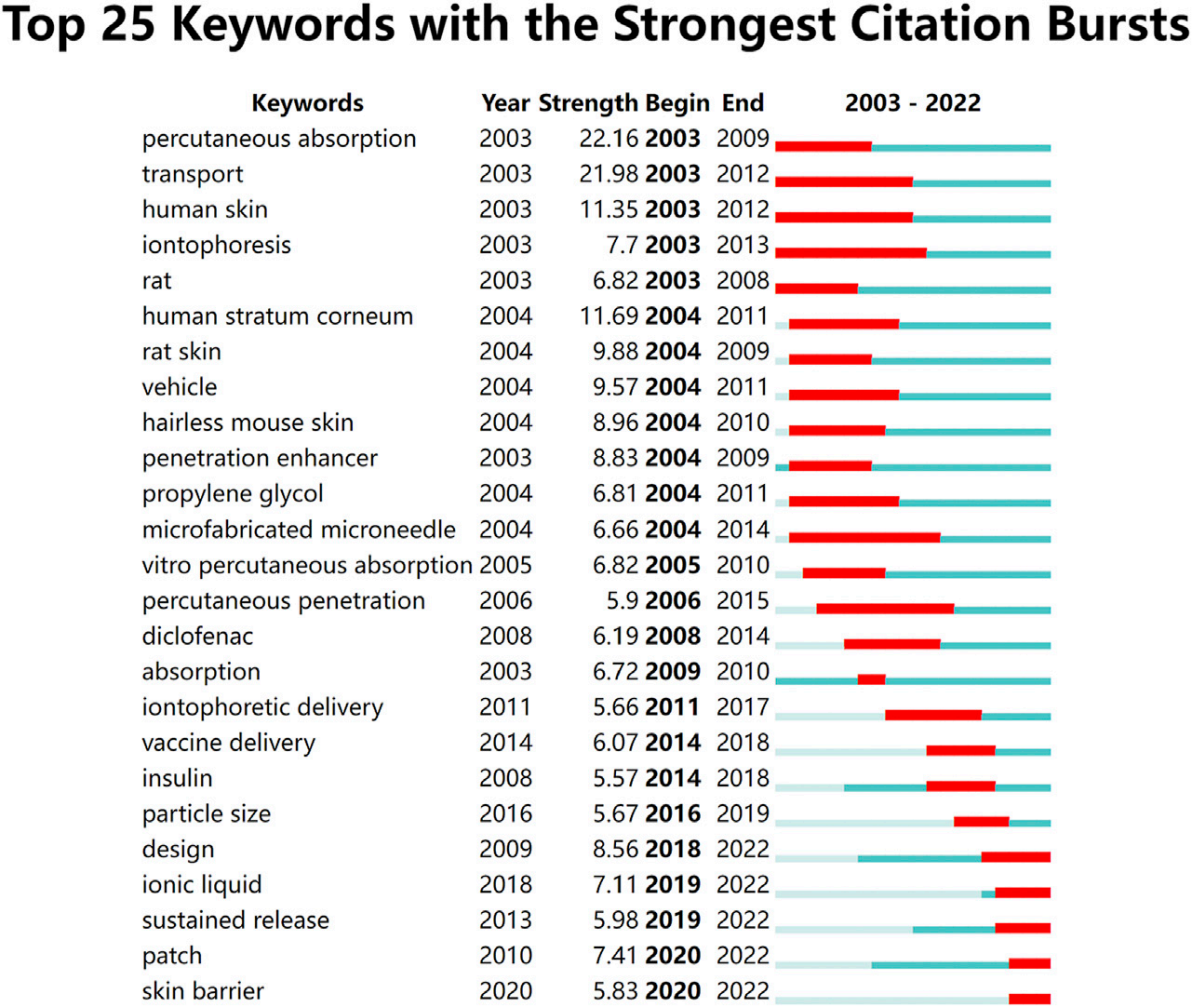
The top 25 burst words from 2003 to 2022.

**FIGURE 9:**
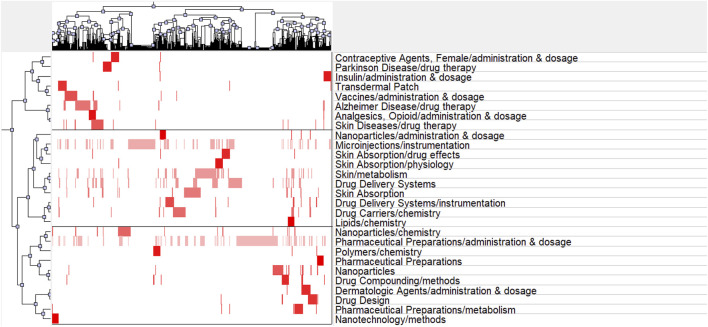
Matrix visualization of major MeSH terms/MeSH subheading terms of articles on transdermal drug delivery.

**FIGURE 10:**
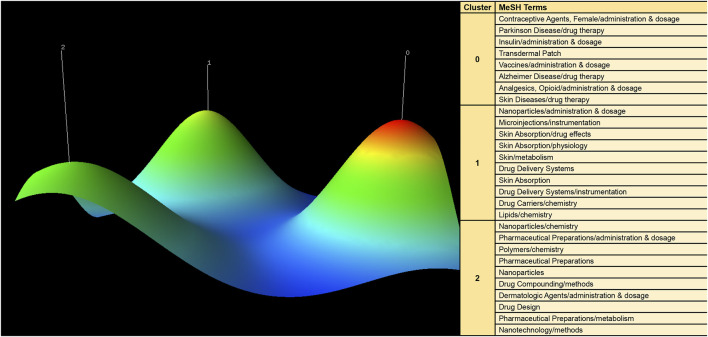
Mountain visualization of major MeSH terms/MeSH subheading terms of articles on transdermal drug delivery.

CiteSpace was utilized to extract keywords from a dataset of 1,536 literature sources, resulting in the identification of the top 25 burst words spanning the period from 2003 to 2022. These burst words elucidate the evolving trends in research hotspots (Figure 8). Additionally, a total of 1,542 major MeSH terms/MeSH subheadings were identified, with a cumulative frequency of 5,559 occurrences. Based on the G-index criterion, high-frequency terms were defined as those appearing more than 10 times (Table 5). To visualize the research hotspots, BICOMB and gCLUTO were employed for biclustering analysis. BICOMB facilitated the establishment of a co-word matrix, which was then imported into gCLUTO for matrix visualization (Figure 9). The left matrix corresponds to the main MeSH terms/MeSH subheading terms depicted on the right. The frequency of terms occurrence was represented by the color of the blocks in the matrix. Subsequently, gCLUTO produced a mountain graph that revealed three distinct clusters within the research field (Figure 10). The distance between mountains denoted the degree of correlation between clusters, while the height and volume of the mountains reflected the internal similarity and the number of covered terms, respectively. Furthermore, the color of the peaks indicated the standard deviation, transitioning from red to green to indicate low to high deviation. Three distinct clusters were derived from the biclustering analysis of the publications (Ⅰ) Drug therapy of transdermal delivery (Ⅱ) Drug delivery of transdermal delivery (Ⅲ) Pharmaceutical preparations and drug design of transdermal delivery.

**TABLE 5:** Major MeSH terms/MeSH subheadings from the included publications on transdermal drug delivery (*n* = 1,542).

Rank	Major MeSH terms/MeSH subheadings	Frequency	Proportion of frequency (%)	Cumulative percentage (%)
1	Drug Delivery Systems	332	5.9723	5.9723
2	Skin/metabolism	159	2.8602	8.8325
3	Pharmaceutical Preparations/administration and dosage	151	2.7163	11.5488
4	Skin Absorption	93	1.673	13.2218
5	Drug Carriers/chemistry	75	1.3492	14.571
6	Nanoparticles	59	1.0613	15.6323
7	Drug Delivery Systems/instrumentation	59	1.0613	16.6936
8	Vaccines/administration and dosage	54	0.9714	17.665
9	Nanoparticles/chemistry	51	0.9174	18.5824
10	Skin Diseases/drug therapy	39	0.7016	19.284
11	Transdermal Patch	39	0.7016	19.9856
12	Dermatologic Agents/administration and dosage	36	0.6476	20.6332
13	Skin Absorption/drug effects	35	0.6296	21.2628
14	Parkinson Disease/drug therapy	33	0.5936	21.8564
15	Insulin/administration and dosage	31	0.5577	22.4141
16	Skin Absorption/physiology	30	0.5397	22.9538
17	Polymers/chemistry	30	0.5397	23.4935
18	Microinjections/instrumentation	28	0.5037	23.9972
19	Analgesics, Opioid/administration and dosage	26	0.4677	24.4649
20	Pharmaceutical Preparations	26	0.4677	24.9326
21	Lipids/chemistry	26	0.4677	25.4003
22	Nanoparticles/administration and dosage	26	0.4677	25.868
23	Alzheimer Disease/drug therapy	22	0.3958	26.2638
24	Drug Design	20	0.3598	26.6236
25	Pharmaceutical Preparations/metabolism	20	0.3598	26.9834
26	Contraceptive Agents, Female/administration and dosage	17	0.3058	27.2892
27	Drug Compounding/methods	13	0.2339	27.5231
28	Nanotechnology/methods	12	0.2159	27.739

## 5 Discussion

The results of the bibliometric analysis indicated that there has been a significant increase in the body of literature on transdermal drug delivery from 2003 to 2022, making it challenging to keep pace with the research hotspots. To address this challenge, a systematic analysis was performed utilizing bibliometric and biclustering analysis techniques. The study was carried out by extracting relevant studies from WOS and NCBI databases, covering a 20-year time span. The results of this analysis are expected to provide valuable insights into the research hotspots and predict future trends.

### 5.1 Overview of transdermal drug delivery research

The current study aimed to evaluate the national academic contributions and quality in the field of transdermal drug delivery by analyzing the number of studies, total number of citations, centrality, and average number of citations produced by the country/region. The results showed that the United States has the most significant impact on transdermal drug delivery research, with a total of 21,847 citations, followed by India with 11,589 citations and China with 11,155 citations. However, the trend of transdermal drug delivery research in the United States showed a decline, with the number of studies reaching a peak in 2020 (69) before decreasing. Conversely, China continued to experience an increase in the number of transdermal drug delivery publications, surpassing the United States in 2014. Although China has a higher number of transdermal drug delivery publications, its impact remains insufficient (centrality = 0.13) and ranks fourth among the top 10 countries. Additionally, the quality of transdermal drug delivery publications in China should be improved, as the average number of citations per study is relatively low (22.72) and ranks eighth among the top 10 countries. The thermal world map also highlights regions with a greater investment in transdermal drug delivery research efforts. The results indicate that regions such as Africa, the Middle East, and Eastern Europe have limited transdermal drug delivery research and may require international cooperation and support to enhance their contributions to the field.

The results of the analysis indicated that over half of the top 10 institutions contributing to the field of transdermal drug delivery belong to the United States (*n* = 3) and Egypt (*n* = 3), demonstrating their substantial impact. A colorful density visualization was utilized to present the clusters of different institutions and to intuitively determine the cooperative relationships between them. Institutions that collaborate closely are represented by the same color cloud, and the larger the cluster area, the more significant the group’s contributions to the field are. Additionally, the size of the institution’s name represents its level of cooperation with other institutions. Universities such as Mercer University, Cairo University, Queen’s University Belfast, and the University of California System were found to have made notable contributions to transdermal drug delivery research.

The leading journals in the field of transdermal drug delivery, namely, the International Journal of Pharmaceutics, Journal of Controlled Release, and Drug Development and Industrial Pharmacy, have collectively accounted for a substantial portion (more than 15%) of the studies published on the subject. The International Journal of Pharmaceutics has established itself as the premier journal in its field, as evidenced by its significantly higher number of transdermal drug delivery studies published compared to its closest competitor. Furthermore, the journal boasts the highest H-index among the top 10 journals, further solidifying its position as a key contributor to the advancement of knowledge in the field of transdermal drug delivery. These findings demonstrate the important role that the International Journal of Pharmaceutics has played in shaping the discourse and advancing the field of transdermal drug delivery.

The study finds that the research hotspot has undergone a shift in its focus between 2009 and 2011, as demonstrated by the analysis of burst words in the field. Prior to this period, the prominent research topics included transportation, microneedles, and percutaneous penetration. Conversely, the study reveals that the current research hotspot revolves around vaccine delivery, insulin, and sustained drug release. It is important to note that this shift in focus does not indicate a decline in research interest in the previous areas of study.

The research focus in transdermal drug delivery has shifted from primarily investigating mechanisms and factors affecting percutaneous absorption, such as penetration enhancers and microneedles, to a broader range of drug delivery and control release methods, including sustained release and patch design, as well as a wider range of research subjects, such as vaccines and insulin. This shift in research focus reflects an increasing emphasis on drug delivery and control release, rather than simply absorption and penetration, and suggests a growing interest in engineering approaches to transdermal drug delivery.

### 5.2 Three clustering hotspots of transdermal drug delivery research

#### 5.2.1 Drug therapy of transdermal drug delivery

Cluster 0 presents a correlation with drug therapy of transdermal delivery. Transdermal drug delivery have been successfully used in the treatment of several different conditions. In the area of vaccine delivery, transdermal drug delivery has been shown to improve efficacy and reduce the incidence of side effects compared to traditional injection methods (Suradhat et al., 2016; Zhu et al., 2020; Rajput et al., 2021; Dewangan and Tomar, 2022). This is particularly relevant for vaccines that require frequent administration, as transdermal drug delivery provides a convenient and painless alternative (Bhowmik et al., 2011; Ita, 2016b; Larraneta et al., 2016; An and Liu, 2017; Wang et al., 2021). Transdermal drug delivery has also shown promising results in insulin delivery for diabetes patients, offering a convenient and effective alternative to traditional insulin injections (Xie et al., 2015; Lee et al., 2017a; Wang et al., 2020; Zhang et al., 2021). Transdermal drug delivery can enhance insulin delivery efficacy, reduce the likelihood of hypoglycemia, and improve patient compliance and satisfaction. The efficacy of transdermal drug delivery in treating skin diseases, such as psoriasis and eczema, have being explored (Preethi et al., 2021; Bozorg et al., 2022; Li et al., 2022). In hair regrowth treatment, transdermal drug delivery offers the potential for improved efficacy and reduced side effects through direct delivery of drugs to the scalp (Yang et al., 2021; Zhao et al., 2022b).

In addition, transdermal drug delivery have also been investigated for their potential use in delivering contraceptive agents, analgesics, protein and peptide drugs, and as a treatment for bone disorders (Jain et al., 2005; Wu et al., 2017; Defraeye et al., 2020; Sharma et al., 2021; Shin et al., 2022; Yang et al., 2023). Transdermal drug delivery have shown promise in the treatment of cardiovascular diseases, such as stroke and coronary artery disease, as well as neurodegenerative disorders such as Alzheimer’s disease and Parkinson’s disease (Quinn et al., 2015; Appleton et al., 2020; Siafaka et al., 2020; Patel et al., 2021). Furthermore, transdermal drug delivery have been actively researched for their potential use in delivering cancer therapies, offering a minimally invasive option for delivering chemotherapy drugs directly to the site of the tumor (Jiang et al., 2020; Truong et al., 2022). Transdermal drug delivery represents a promising method for delivering various therapeutic agents and have the potential to improve patient outcomes and quality of life. Further research is needed to fully realize the potential of this drug delivery method.

#### 5.2.2 Drug delivery of transdermal drug delivery

Cluster 1 presents the correlations with drug delivery of transdermal delivery. Microneedles are one of the most promising drug delivery technologies for transdermal drug delivery (Zhao et al., 2021). They are small, needle-like structures that can penetrate the skin, but are small enough to avoid causing pain. The microneedles can be made of biodegradable materials, and they always be coated with drugs to improve the skin’s permeation of the drugs (Yu et al., 2017a; Hmingthansanga et al., 2022; Malek-Khatabi et al., 2022). The use of microneedles in transdermal drug delivery has shown promising results in various areas of drug therapy, including insulin delivery for diabetes, vaccine delivery, and pain management.

Chemical Penetration Enhancers (CPEs) are another important technology for transdermal drug delivery (Pathan and Setty, 2009). CPEs are compounds that can improve the skin’s permeation of drugs by altering the stratum corneum, the outermost layer of the skin (Yu et al., 2017b). CPEs can be used alone or in combination with other transdermal drug delivery technologies, such as microneedles and nanocarriers, to enhance drug delivery and improve patient outcomes. Vesicles, nanoemulsions, nanoparticles, and nanocrystals are nanocarriers that can be used to improve transdermal drug delivery (Jain et al., 2003; Wang et al., 2017; Rai et al., 2018; Yu et al., 2018; Cao et al., 2020; Garg et al., 2021; Huang et al., 2021; Chen et al., 2022b). Nanocarriers can be loaded with drugs and are designed to protect the drugs from degradation, enhance their permeation through the skin, and improve their pharmacokinetic and pharmacodynamic properties. Nanocarriers have been evaluated in various areas of drug therapy, including vaccines, skin diseases, hair regrowth, contraceptive agents, analgesics, protein and peptide drugs, and cancer (Fornaguera et al., 2015; Damani et al., 2021; Nooraei et al., 2021; Yang et al., 2021).

Solid dispersions, physical methods for transdermal drug delivery, electrical techniques, high pressure-based devices, mechanical approaches, and the integration of chemical and physical technologies are other important technologies for transdermal drug delivery (Bhaskar et al., 2009; Vitorino et al., 2014; Munch et al., 2017; Pandey et al., 2019). These technologies can be used alone or in combination with other transdermal drug delivery technologies to enhance drug delivery and improve patient outcomes. The development of various drug delivery technologies may be crucial to further improve transdermal drug delivery.

#### 5.2.3 Pharmaceutical preparations and drug design of transdermal drug delivery

Cluster 2 presents a correlation with drug design of transdermal delivery. The design of transdermal drug delivery is a crucial factor in the development of effective transdermal systems. In order to successfully deliver a drug through the skin, it must be able to penetrate the outer layers and reach the target site of action. The design need take into account the properties of the skin, including its thickness, permeability, and the presence of any active transport mechanisms (Alkilani et al., 2018; Lai et al., 2020). Pharmaceutical preparations play an important role in the design of drugs for transdermal drug delivery (Ahsan et al., 2021). The formulation of the drug, the type of transdermal device used, and the skin properties of the individual all have an impact on the efficacy and safety of the system (Kwon et al., 2015; Ashtikar et al., 2016; Lee et al., 2017b). For example, prodrugs are inactive compounds that are designed to be converted into the active drug form after reaching the target site of action (Li et al., 2017). This approach has the advantage of reducing the toxicity of the drug and increasing its efficacy, as well as reducing the need for frequent dosing.

Polymers are commonly used to transdermal drug delivery design. Polymers can be used to create a matrix or reservoir that can store the drug and provide a controlled release over time (Lee et al., 2015; Suksaeree et al., 2018). The chemical property of the drug is also a key factor in the design of transdermal drug delivery (Kalvodova and Zbytovska, 2022). Such as its solubility and stability, can greatly affect its ability to penetrate the skin and reach the target site of action (Lapteva and Kalia, 2013). To improve these properties, drug compounding techniques can be used to modify the drug and increase its effectiveness. Nanotechnology has also been used in transdermal drug delivery research to improve the efficacy and safety of transdermal delivery (Shang et al., 2022).

The administration and dosage of transdermal drug delivery drugs need to be considered in the design process (Roy et al., 2022). The administration site and method should be carefully selected to ensure optimal delivery and to minimize the risk of side effects (Waghule et al., 2019). The dosage also should be carefully optimized to ensure the desired therapeutic effect is achieved (Baek et al., 2013; Liu et al., 2020). The design of drugs for transdermal drug delivery is a complex and multi-faceted process that requires careful consideration of various factors. Further research is needed to optimize drug design of transdermal drug delivery and to develop new and innovative transdermal systems for a wide range of medical conditions.

### 5.3 Limitation

The utilization of bibliometric evaluation for research analysis is subject to certain limitations. One such limitation is the possibility of exclusion of high-quality studies from the data due to insufficient citation frequency. This can occur due to the delayed recognition of recently published studies, as bibliometrics primarily relies on the analysis of publication citation count (Wallin, 2005). Additionally, the biclustering analysis used to classify and identify prominent keywords may not necessarily cover all relevant topics in a field of study. Furthermore, the data collected from the Web of Science database may not include the most current publications due to updating lag times. Despite these limitations, bibliometric analysis remains a valuable tool for gaining a comprehensive and efficient understanding of the research landscape in a particular field.

## 6 Conclusion

The field of transdermal drug delivery has garnered increased attention in recent years, necessitating the need for an analysis of the current research trends. This study provides a summary of key information related to transdermal drug delivery publications, including the number of studies, country of origin, institutions involved, and the journals in which they were published. The results indicate that the United States has made the most significant contribution to transdermal drug delivery research. Additionally, while the number of transdermal drug delivery studies originating from China has seen substantial growth, further improvement in the quality of these studies is required. Despite the ongoing challenges in the field of transdermal drug delivery, such as ensuring consistent drug permeation and improving stability during storage, the future of transdermal drug delivery appears to be promising. Advances in transdermal drug delivery techniques and materials hold significant potential to enhance the efficacy and stability of transdermal systems. Therefore, it is expected that further research will develop more reliable and effective transdermal drug delivery systems that ultimately benefit patient outcomes. Hopefully, this study can provide guidance for global researchers.

## Data Availability

The original contributions presented in the study are included in the article/supplementary material, further inquiries can be directed to the corresponding authors.
